# Uniparental disomy determined by whole‐exome sequencing in a spectrum of rare motoneuron diseases and ataxias

**DOI:** 10.1002/mgg3.285

**Published:** 2017-04-05

**Authors:** Dana M. Bis, Rebecca Schüle, Jennifer Reichbauer, Matthis Synofzik, Tim W. Rattay, Anne Soehn, Peter de Jonghe, Ludger Schöls, Stephan Züchner

**Affiliations:** ^1^ John P. Hussman Institute for Human GenomicsUniversity of MiamiMiamiFlorida; ^2^ Dr. John T. Macdonald Foundation Department of Human GeneticsUniversity of MiamiMiamiFlorida; ^3^ Center for Neurology and Hertie Institute for Clinical Brain ResearchEberhard‐Karls‐UniversityTübingenGermany; ^4^ German Center of Neurodegenerative Diseases (DZNE)TübingenGermany; ^5^ Department of Medical GeneticsInstitute of Human GeneticsUniversity of TübingenTübingenGermany; ^6^ Neurogenetics GroupDepartment of Molecular GeneticsVIBUniversity of AntwerpCampus Drie Eiken, Universiteitsplein 1Antwerp2610Belgium; ^7^ Department of NeurologyAntwerp University HospitalAntwerpBelgium; ^8^ Laboratories of Neurogenetics and NeuropathologyInstitute Born‐BungeUniversity of AntwerpUniversiteitsplein 1Antwerp2610Belgium

**Keywords:** Ataxia, motoneuron disease, uniparental disomy, whole exome

## Abstract

**Background:**

The genetic causes of many rare inherited motoneuron diseases and ataxias (MND and ATX) remain largely unresolved, especially for sporadic patients, despite tremendous advances in gene discovery. Whole exome data is often available for patients, but it is rarely evaluated for unusual inheritance patterns, such as uniparental disomy (UPD). UPD is the inheritance of two copies of a chromosomal region from one parent, which may generate homozygosity for a deleterious recessive variant from only one carrier‐parent. Detection of UPD‐caused homozygous disease‐causing variants is detrimental to accurate genetic counseling. Whole‐exome sequencing can allow for the detection of such events.

**Methods:**

We systematically studied the exomes of a phenotypically heterogeneous cohort of unresolved cases (*n* = 96 families) to reveal UPD events hindering a diagnosis and to evaluate the prevalence of UPD in recessive MND and ATX.

**Results:**

One hereditary spastic paraplegia case harbored homozygous regions spanning 80% of chromosome 16. A homozygous disease‐causing mutation in the SPG35 disease gene was then identified within this region.

**Conclusion:**

This study demonstrates the ability to detect UPD in exome data of index patients. Our results suggest that UPD is a rare mechanism for recessive MND and ATX.

## Introduction

Despite the advances whole‐exome sequencing (WES) has brought to clinical genetics, a large portion of cases remains unresolved (Lee et al. 2014). Complicated modes of inheritance, such as uniparental disomy (UPD), can obscure a diagnosis under standard WES analysis. Here, we demonstrate the ability to evaluate an index patient's WES data for UPD, and interrogate the prevalence of UPD within sporadic inherited motoneuron disease and ataxia cases (MND and ATX).

UPD is the inheritance of both chromosomal homologs from one parent (Robinson 2000). The affected region can be confined to a portion of the chromosome (segmental) or can span the entire chromosome (complete), and is an identical copy of one parental allele (isodisomy) or distinct alleles from the same parent (heterodisomy) (King et al. 2014). Isodisomy can generate homozygosity of a deleterious recessive mutation from a heterozygous carrier‐parent (Papenhausen et al. 2011). Both clinical and research settings are impacted by undiagnosed isodisomy. Genetic counseling for the proband's parents would not be accurate without knowledge of the isodisomic event. Isodisomy would also cause incongruent segregation analysis. In a research laboratory, where Sanger‐confirmed segregation analysis is standard, an incongruent result will require time and resources to resolve. Accordingly, an accessible method to investigate these events is increasingly important to reach a diagnosis. Due to the contiguous stretches of homozygosity created by isodisomy, it is now detectable from single nucleotide polymorphism‐based (SNP) data (Papenhausen et al. 2011). A novel computational algorithm, H^3^M^2^, has been adapted for the sparse, irregular SNP distribution of WES (Magi et al. 2014). In this study, we evaluate the feasibility of applying the H^3^M^2^ algorithm to the detection of UPD based on index WES data.

Additionally, we were interested in the prevalence of isodisomy as the disease mechanism within apparently sporadic MND and ATX as it has already been reported in over 25 recessive diseases (Zlotogora 2004). The commonly quoted frequency of UPD within the general population (one in 3500 births) is largely driven by the cases of imprinting disorders that are brought to clinical attention (Robinson 2000). However, UPD occurrence within the Deciphering Developmental Disorders project was recently reported to be almost 20‐fold higher (six in 1057 trios), indicating that UPD frequencies are variable and disease‐specific (King et al. 2014).

## Material and Methods

### Ethical compliance

The patients were collected from the University of Antwerp, the University Hospital Tübingen, and the University of Miami, and all participating individuals gave informed consent prior to initiating this study in agreement with the institutional review boards.

### Patient inclusion

We studied 96 unrelated cases from simplex families with a spectrum of rare neurodegenerative disorders from the ataxia‐motoneuron spectrum and no reported consanguinity. Four consanguineous index cases were additionally included in the study as a positive control for the detection method. The cohort was derived from unresolved cases in the GENESIS database and included the following phenotypes: early onset ataxia (50 index cases), hereditary spastic paraplegias (35 index cases), and amyotrophic lateral sclerosis/frontotemporal dementia (11 index cases) (Figure S1) (Gonzalez et al. 2015).

### Whole‐exome sequencing and variant analysis

Whole‐exome sequencing was performed at the John P. Hussman Institute for Human Genomics at the University of Miami. Samples were enriched with the SureSelect Human All Exon 50 Mb kit (Agilent, Santa Clara, CA), underwent standard sample preparation for the Illumina Hiseq2000 platform and were processed using the GENESIS/GEM.app analysis pipeline (Gonzalez et al. 2015). BAM files were retained as input for the H^3^M^2^ isodisomy mapping. Each putative isodisomic region was closely investigated for high quality (read depth ≥10 and genotype quality ≥35), rare (1000 Genomes Project minor allele frequency ≤0.01 and Exome Aggregation Consortium ≤0.01), homozygous, pathogenic variants within known disease genes gathered from the Genetic Testing Registry and Online Mendelian Inheritance in Man (see interrogated genes in Table S1) (Genomes Project C et al. 2015; Lek et al. 2016).

### Isodisomy mapping

Regions of homozygosity were detected using H^3^M^2^ with recommended parameters (DNorm = 100000, P1 = 0.1, P2 = 0.1). This algorithm takes a BAM file as input to calculate the ratio between the alternate allele reads and the total coverage at each polymorphic position (based on the 1000 Genomes Project). This ratio is used to determine the genotype state at each SNP. It is a discrete state Hidden Markov Model that includes a parameter to account for sequencing and alignment errors and a parameter within the transition probabilities matrix to account for the inconsistent distance between SNPs within WES (Magi et al. 2014).

## Results

### Isodisomy mapping and uniparental disomy confirmation

**Figure 1:**
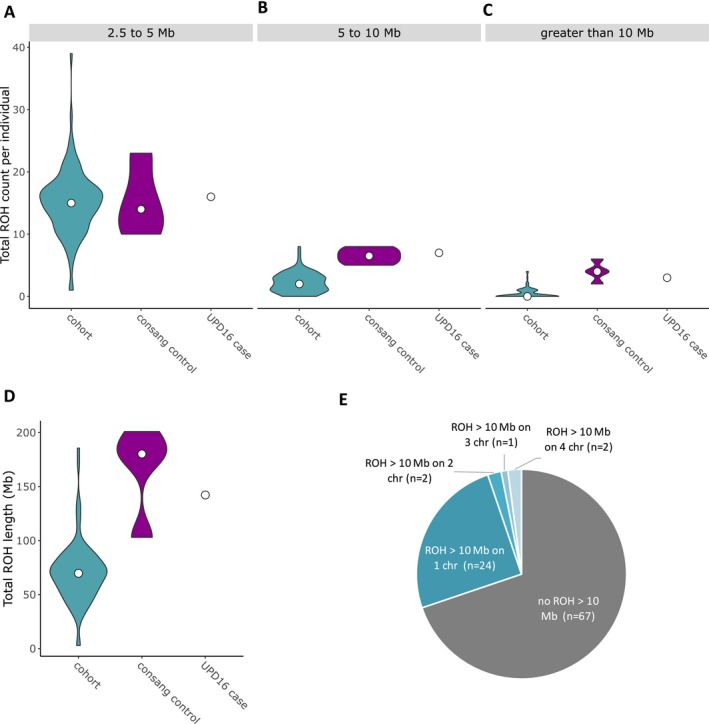
Data are represented as “violin plots,” depicting the distribution of ROH counts per individual [(A) 2.5–5 Mb, (B) 5–10 Mb, and (C) >10 Mb] and the total ROH length (D) within the entire cohort (excluding the UPD16 case), the consanguineous controls, and the identified UPD16 case. The width of each “violin” represents the 90°‐rotated kernel density trace and its reflection and the white dot shows the median. The “pie chart,” (E) displays the distribution of cases within the cohort by the amount of ROH >10 Mb each contains.

**Figure 2:**
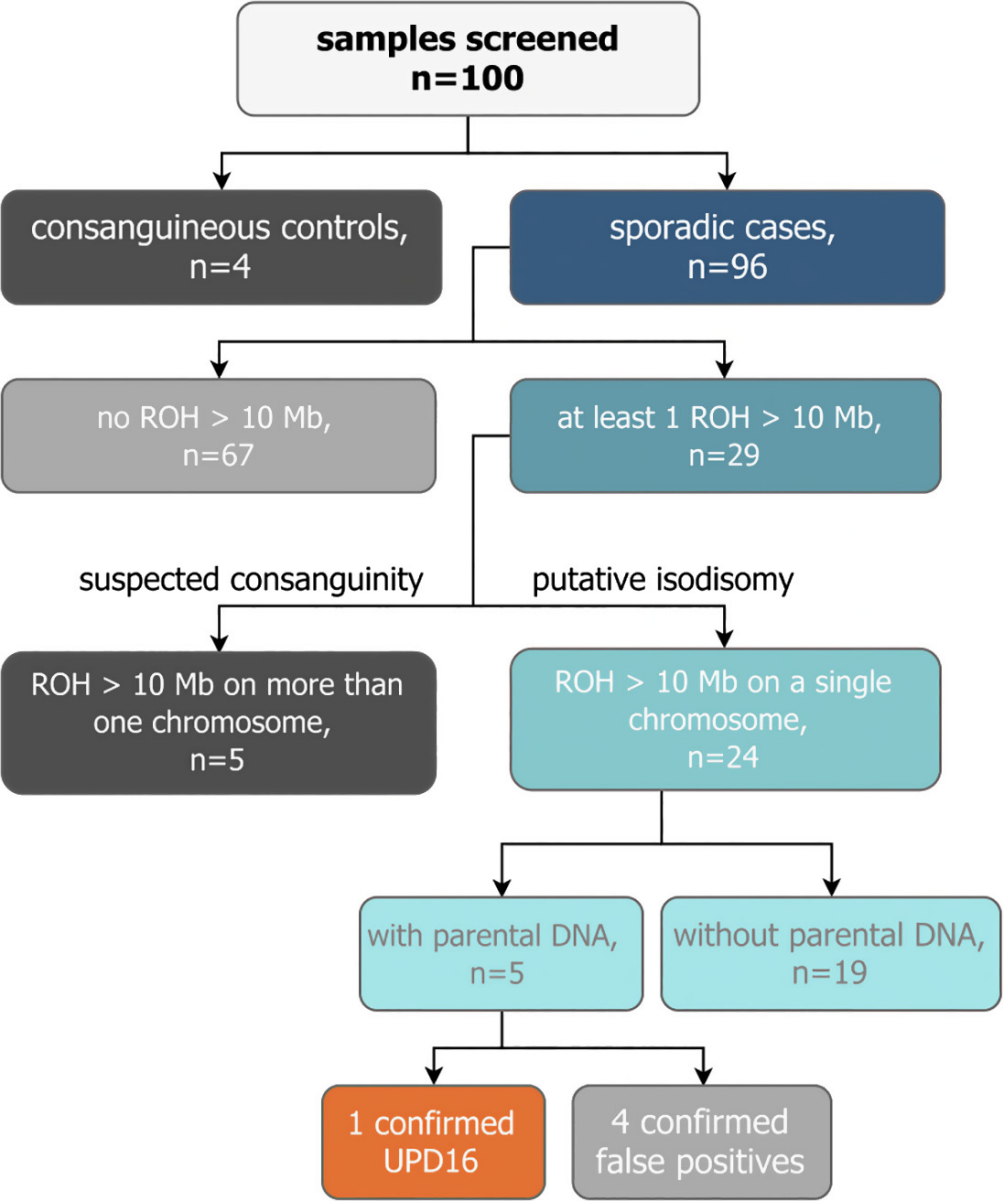
Flowchart describing UPD screening in 96 sporadic cases and four consanguineous controls. Gray indicates consanguinity and false positives, blue indicates possible UPD, and orange highlights confirmed UPD.

We analyzed 96 patients from non‐consanguineous, simplex families with inherited motoneuron disease and ataxia (MND and ATX) to identify potential isodisomic events. The H^3^M^2^ algorithm was applied to each patient's BAM file to detect long regions of homozygosity. Such extended homozygous regions could indicate uniparental isodisomy of a chromosome. Based on standard clinical analysis, homozygous regions below 2.5 Mb were filtered from the analysis (Papenhausen et al. 2011). We used a minimum ROH length threshold to improve the signal‐to‐noise ratio for cohort‐level distribution summaries. All cases contained at least one homozygous region >2.5 Mb (Fig. 1A). Similar to previously published strategies, we defined “putative isodisomy” as the presence of a homozygous region over 10 Mb on just one chromosome (Papenhausen et al. 2011). From the screened cohort, 29 cases contained at least one homozygous region >10 Mb. Of these, five contained homozygous regions on multiple chromosomes, indicating hidden consanguinity. The remaining 24 cases contained one or more homozygous regions over 10 Mb on a single chromosome (Fig. 1E) and were thus classified as putative isodisomy cases. To validate the putative UPD regions we selected high quality (read depth ≥10 and genotype quality ≥75), rare (1000 Genomes Project minor allele frequency ≤0.001 and Exome Aggregation Consortium ≤0.001), homozygous variants within the possible UPD site for Sanger‐based segregation analysis in five cases with available parental DNA (Fig. 2) (Genomes Project C et al. 2015; Lek et al. 2016). Additionally, parental DNA of two cases with consanguineous family background and one case without reported consanguinity yet multiple runs of homozygosity (ROH) >10 Mb were available for genotyping.

**Figure 3:**
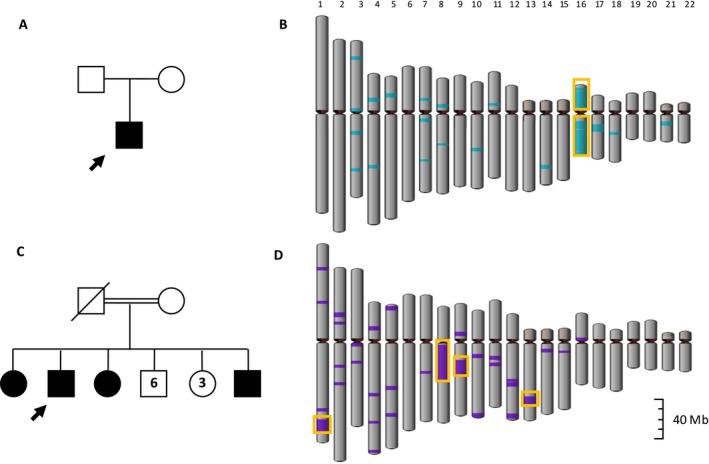
Isodisomy mapping of (A) the UPD16 case and (B) a consanguineous control. All ROH >2.5 Mb are shown with a color overlay and ROH >10 Mb our highlight in yellow. (A) displays the complete isodisomic event on chromosome 16, while (B) shows multiple ROH >10 Mb dispersed to multiple chromosomes.

The isodisomic mapping revealed a strong signal on chromosome 16 comprised of multiple large ROHs which sum to 72 Mb and cover 80% of the chromosome in one HSP case (SPG35, MIM #612319) (Fig. 3A,B), indicating putative complete isodisomy. During the same time as this study, this case was undergoing independent investigation for UPD at the University Hospital Tübingen. UPD was molecularly verified by excluding genomic deletions with multiplex ligation‐dependent probe amplification assay and performing fragment analysis of PCR amplicons of microsatellite marks within the index patient and both parents. A homozygous p.Trp176* mutation was identified within *FA2H* (MIM *611026, NM_024306.4), a known SPG35 disease gene. Sanger sequencing confirmed that only the father was a carrier of the identified variant (Soehn et al. 2016).

In the remaining four cases with putative UPD and available parental DNA, segregation analysis was not consistent with UPD (Table 1). After closer inspection of these four cases, case 01 contains a 9.1 Mb ROH on a separate chromosome. Though this ROH is below our defined UPD threshold, it may indicate hidden consanguinity. All consanguineous controls contained homozygous regions >10 Mb on multiple chromosomes as expected (Fig. 3C,D). Segregation analysis of each ROH >10 Mb was performed on two consanguineous controls and one suspected consanguineous case with available parental DNA. Tested SNVs within the ROHs of both consanguineous controls and suspected consanguineous case were compatible with recessive inheritance.

**Table 1:** Segregation analysis for five sporadic cases with possible UPD based on the presence of a homozygous region >10 Mb isolated to a single chromosome

Case	Homozygous	Region			Tested variant	Index	Father	Mother	Conclusion
	Chr	Start	End	Size (Mb)					
01	1	192312018	203317394	11.0	Chr1:202698171C/T	T/T	C/T	C/T	Recessive inheritance
02	12	93964833	109547815	15.6	Chr12:108912996G/A	A/A	G/A	G/A	Recessive inheritance
03	13	61013944	72440942	11.4	Chr13:71725803G/A	A/A	G/A	G/A	Recessive inheritance
04	13	80910758	92101290	11.2	Chr13:80940229G/A	A/A	G/A	G/A	Recessive inheritance
UPD16	16	70284990	83842988	13.6	Chr16:74760209C/T	T/T	C/T	C/C	Compatible with UPD

Notably, we identified a region enriched for ROH on the q arm of chromosome 13, a gene poor region (average gene density below three genes per Mb) from 52.9–90.7 Mb (Dunham et al. 2004). ROHs in this region were present in a total of 18 index cases in our cohort: 13 of 24 putative UPD (2 with parental DNA), three of five suspected consanguinity, and two of four consanguineous controls (Figure S2). The three cases with suspected consanguinity still contain ROH on multiple chromosomes even if the chromosome 13q ROH are removed. Segregation analysis of ROHs within this region was possible in three samples (cases 03 and 04 in Table 1; one consanguineous control), and revealed that the ROH were not compatible with UPD. The remaining nine cases with putative UPD but without parental DNA contained ROH on separate chromosomes (Figure S3).

## Discussion

Even though WES is a powerful tool for diagnosing genetic diseases, approximately 75% of cases remain undiagnosed after WES analysis (Lee et al. 2014). In order to increase the amount of diagnoses, WES should be analyzed for rare disease‐causing mechanisms whenever possible. In this study, we applied the novel H^3^M^2^ algorithm to an index patient's WES and successfully detected complete uniparental isodisomy. Because of H^3^M^2^'s basic hardware requirements and quick computational speed, it can rapidly be used on existing WES or readily incorporated into an exome analysis pipeline. Incorporation of such a ROH detection tool into an exome analysis pipeline is preferable to manual detection as it allows for objective, scalable, and high‐throughput results while accounting for inconsistent distances between SNPs. H^3^M^2^ achieves more accurate ROH identification by including a parameter which accounts for sequencing and alignment errors. Additionally, H^3^M^2^ is independent of genotype‐caller errors as it calculates the *B*‐allele frequency directly from the alignment file. Isodisomy mapping is a powerful technique as it provides a simple way to extract information critical to a diagnosis solely from a proband's WES. This method is particularly equipped to detect complete isodisomy from a single exome; however, due to small segmental background homozygosity in the general population (Pemberton et al. 2012), it does not fully capture small segmental isodisomies without trios analysis. Though trio studies are the most informative analysis for all uniparental disomies, they are not always available or financially practical. Therefore, isodisomy mapping provides efficient means of distinguishing, which cases warrant further confirmation of uniparental isodisomy from already available patient data.

The criteria for defining a putative isodisomy event will likely need to be adjusted to improve the rate of true positives. In addition to homozygous regions over 10 Mb on a single chromosome, the cumulative percentage of homozygous regions across a chromosome may be considered. Although the H^3^M^2^ algorithm is designed to handle the non‐uniform distribution of the whole exome, our experience indicates that gene poor regions may produce false positive calls for UPD. Half of the detected putative UPD cases (*n* = 12/24) were located within a gene poor region on chromosome 13q. Segregation analysis did not support UPD in two of these cases (Table 1). We suspect these ROH are primarily generated due to unusually long non‐informative regions of the genome. In this study, we identified the chromosome 13q region initially by the high frequency of ROH within the region and followed by a literature search. We did not observe additional regions with high ROH frequency suggestive of a false‐positive region. As our experience with this method grows, this and possibly other gene poor regions, will likely have to be excluded from analysis.

Beyond detection of complete isodisomy, we were also able to identify potential hidden consanguinity in approximately 5% of our cohort. Hidden consanguinity is not usually addressed during WES analysis as it is not considered to be a frequent event. Our results challenge this standard view and indicate that distant consanguinity may occur more often than expected. As such, this approach additionally helps to unravel hidden consanguinity within cases without a known family history.

We sought to evaluate the prevalence of UPD within a cohort of recessively inherited motoneuron diseases and ataxias (MND and ATX). Only complete isodisomy was considered for the prevalence analysis, as this screening method is not well suited for detection of small segmental isodisomies. We observed one complete isodisomy within 96 screened cases (1.04%) which is not significantly different from the four complete isodisomies observed within the Deciphering Developmental Disorders (DDD) cohort^2^ (*P*‐value is 0.356 [Fischer's exact test]). However, like the DDD cohort, our cohort is significantly different than the general UPD rate of one in 3500 births^1^ (*P*‐value is 0.0267 [binomial test]). Our results suggest that complete isodisomy occurs more frequently in recessively inherited MND and ATX than in the general population. Notably, this frequency will likely increase when disease‐causing segmental isodisomies are considered. However, considering the recently reported cases of UPD in spastic paraplegia type 35 cases, it is unclear whether our observed frequency is driven by a SPG35‐specific phenomenon (Soehn et al. 2016).

In summary, isodisomy mapping can detect complete isodisomy from WES and can be easily implemented into a bioinformatics workflow. Clinically, isodisomy should be especially suspected in cases with homozygous variants in the absence of a consanguineous family history and prompt further analysis. Currently, complete isodisomy appears to be a rare mechanism of disease in recessively inherited MND and ATX. This topic should be revisited as maximum resolution of these rare events, including segmental isodisomies, is attained through whole genome sequencing.

## Disclosure

No conflicts of interest to report.

## Supporting information


**Figure S1.** Phenotypic summary of studied cohort including Amyotrophic lateral sclerosis with frontotemporal dementia (ALS/FTD), inherited ataxias (ATX), complicated hereditary spastic paraplegia (cHSP) and pure hereditary spastic paraplegia (pHSP).

**Figure S2.** Chromosome 13 region showing (A) the previously reported gene poor region across chromosome 13 (in orange), (B) homozygous regions >10 Mb; blue regions are sporadic index cases, gray regions are consanguineous control index cases; (C) UCSC hg19 Known Gene track.

**Figure S3.** Location of putative isodisomic regions for cases without parental DNA which did not fall into chromosome 13q gene‐poor region. Each colored box represents an independent patient.

**Table S1.** Detailed information about the genes analyzed for each phenotype within the cohort.
