# Supplementary figures and images for: Uniparental disomy determined by whole‐exome sequencing in a spectrum of rare motoneuron diseases and ataxias
Source: Mol Genet Genomic Med. 2017 Apr 5;5(3):280–6. doi: 10.1002/mgg3.285 (PMC5441426; doi:10.1002/mgg3.285)

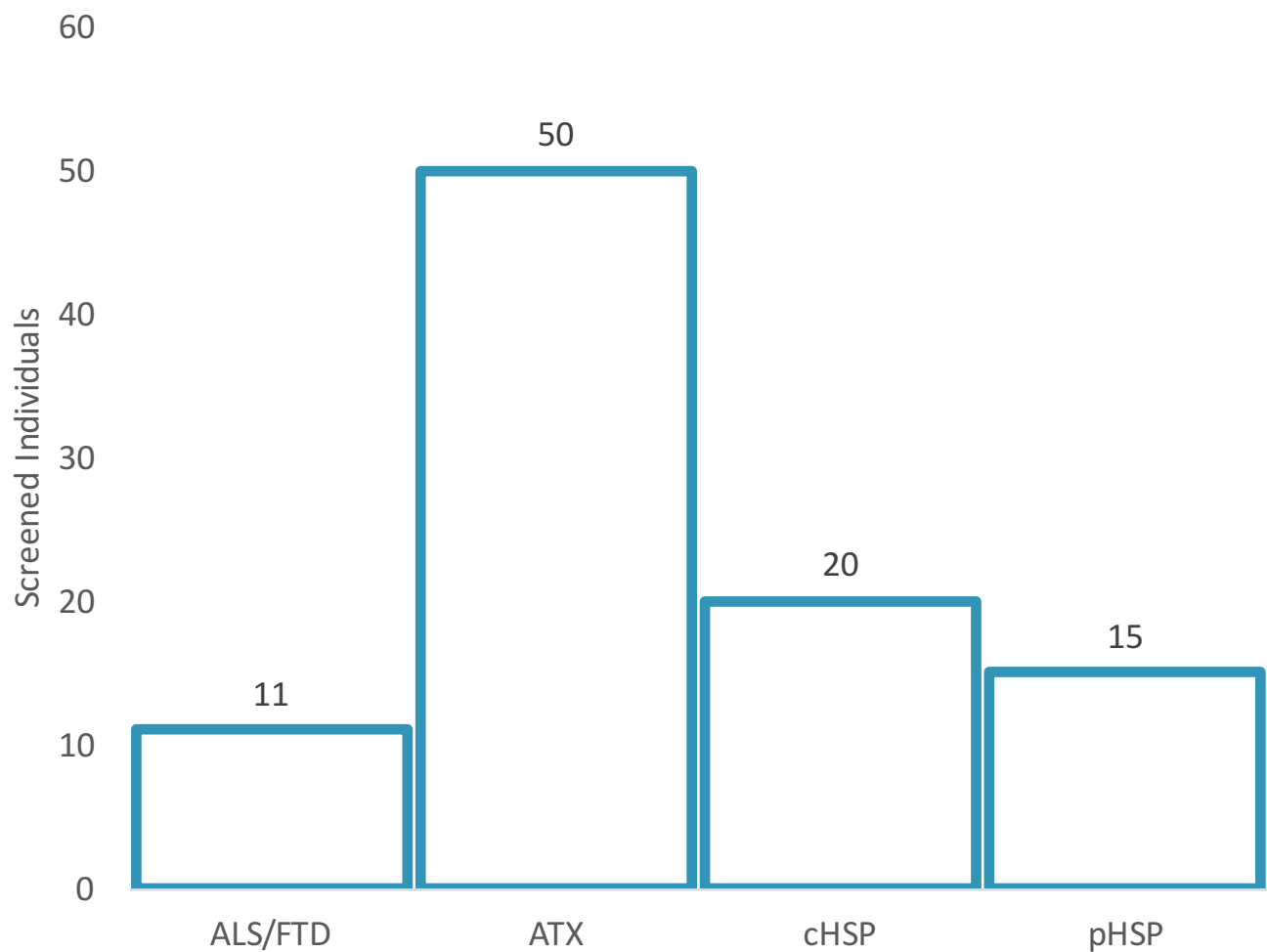

Supplement: Supplementary file 1 — Figure S1. Phenotypic summary of studied cohort including Amyotrophic lateral sclerosis with frontotemporal dementia (ALS/FTD), inherited ataxias (ATX), complicated hereditary spastic paraplegia (cHSP) and pure hereditary spastic paraplegia (pHSP). [file MGG3-5-280-s001.pdf]

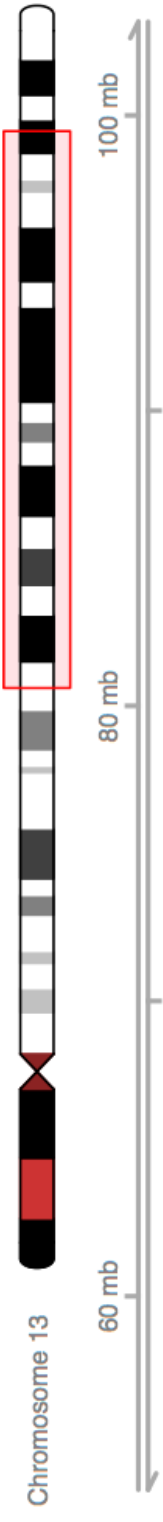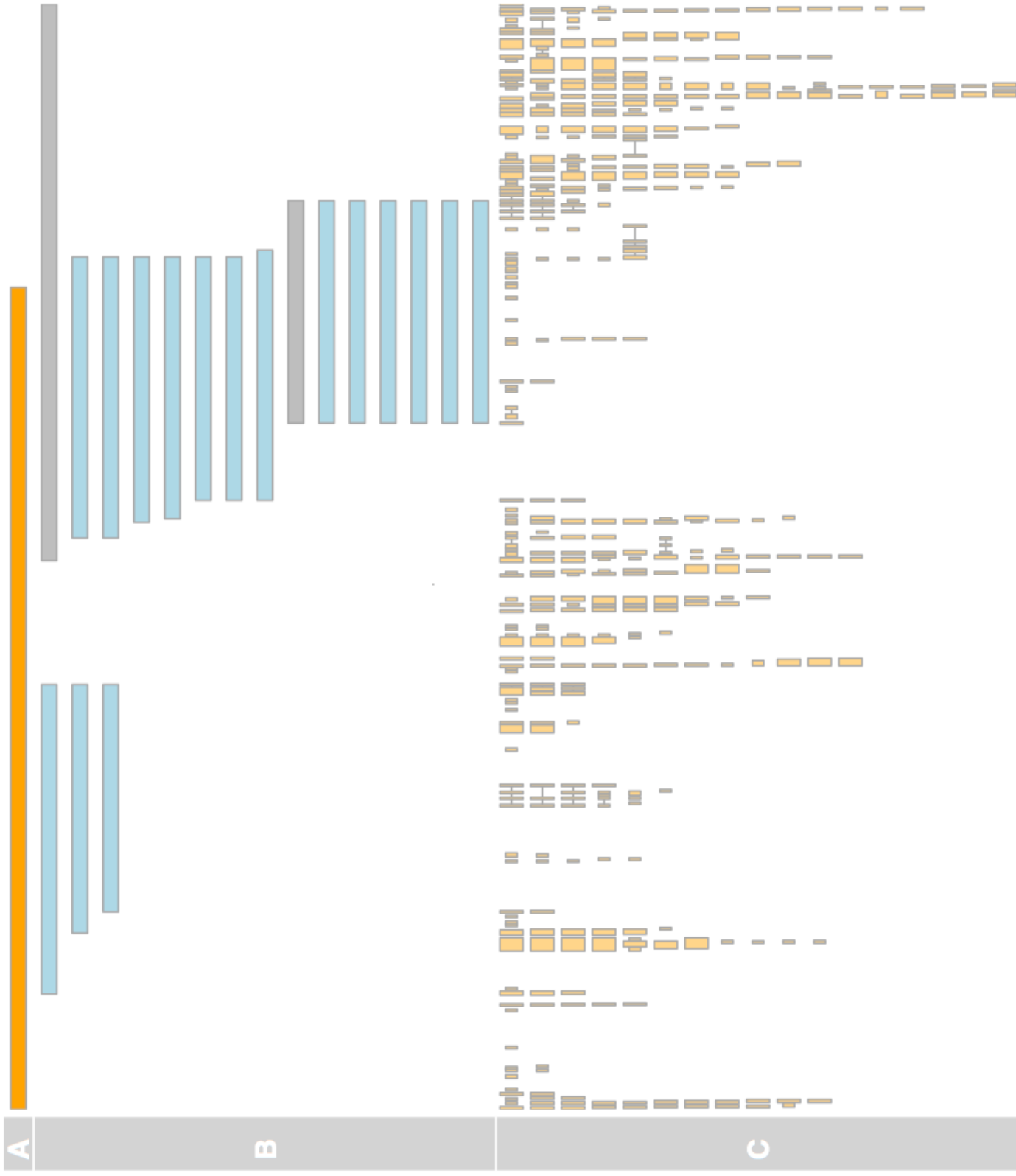

Supplement: Supplementary file 2 — Figure S2. Chromosome 13 region showing (A) the previously reported gene poor region across chromosome 13 (in orange), (B) homozygous regions >10 Mb; blue regions are sporadic index cases, gray regions are consanguineous control index cases; (C) UCSC hg19 Known Gene track. [file MGG3-5-280-s002.pdf]

## Unconfirmed putative isodisomy cases

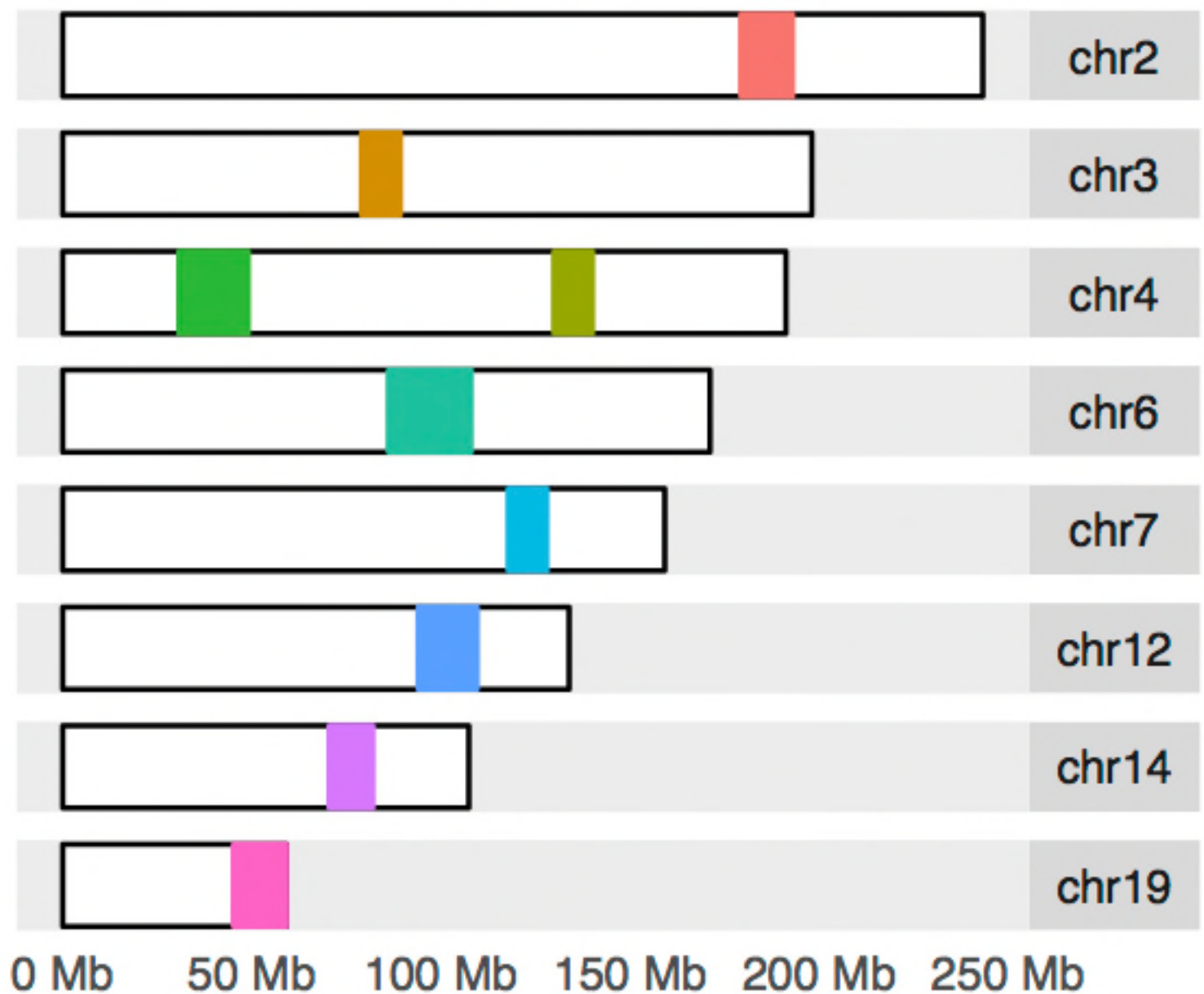

Supplement: Supplementary file 3 — Figure S3. Location of putative isodisomic regions for cases without parental DNA which did not fall into chromosome 13q gene‐poor region. Each colored box represents an independent patient. [file MGG3-5-280-s003.pdf]
