# Supplementary material for: Uniparental disomy determined by whole‐exome sequencing in a spectrum of rare motoneuron diseases and ataxias
Source: Mol Genet Genomic Med. 2017 Apr 5;5(3):280–6. doi: 10.1002/mgg3.285 (PMC5441426; doi:10.1002/mgg3.285)
Supplement: Supplementary file 4 — Table S1. Detailed information about the genes analyzed for each phenotype within the cohort. [file MGG3-5-280-s004.docx]

Supplemental Table 1

| **Gene Symbol** | **Genomic Locus** | **MIM Number** | **Genetic Registry Testing ID** | **Phenotype** |
| --- | --- | --- | --- | --- |
| ANG | 14q11.2 | 105850 | GTR000523316.8 | Amyotrophic Lateral Sclerosis and Frontotemporal Dementia |
| ARHGEF28 | 5q13.2 | 612790 | GTR000523316.8 | Amyotrophic Lateral Sclerosis and Frontotemporal Dementia |
| C9orf72 | 9p21.2 | 614260 | GTR000523316.8 | Amyotrophic Lateral Sclerosis and Frontotemporal Dementia |
| CDH13 | 16q23.3 | 601364 | GTR000523316.8 | Amyotrophic Lateral Sclerosis and Frontotemporal Dementia |
| CHMP2B | 3p11.2 | 609512 | GTR000523316.8 | Amyotrophic Lateral Sclerosis and Frontotemporal Dementia |
| FUS | 16p11.2 | 137070 | GTR000523316.8 | Amyotrophic Lateral Sclerosis and Frontotemporal Dementia |
| GRN | 17q21.31 | 138945 | GTR000523316.8 | Amyotrophic Lateral Sclerosis and Frontotemporal Dementia |
| HNRNPA1 | 12q13.13 | 164017 | GTR000523316.8 | Amyotrophic Lateral Sclerosis and Frontotemporal Dementia |
| HNRNPA2B1 | 7p15.2 | 600124 | GTR000523316.8 | Amyotrophic Lateral Sclerosis and Frontotemporal Dementia |
| MAPT | 17q21.31 | 157140 | GTR000523316.8 | Amyotrophic Lateral Sclerosis and Frontotemporal Dementia |
| OPTN | 10p13 | 602432 | GTR000523316.8 | Amyotrophic Lateral Sclerosis and Frontotemporal Dementia |
| PFN1 | 17p13.2 | 176610 | GTR000523316.8 | Amyotrophic Lateral Sclerosis and Frontotemporal Dementia |
| PSEN1 | 14q24.2 | 104311 | GTR000523316.8 | Amyotrophic Lateral Sclerosis and Frontotemporal Dementia |
| PSEN2 | 1q42.13 | 600759 | GTR000523316.8 | Amyotrophic Lateral Sclerosis and Frontotemporal Dementia |
| SOD1 | 21q22.11 | 147450 | GTR000523316.8 | Amyotrophic Lateral Sclerosis and Frontotemporal Dementia |
| SQSTM1 | 5q35.3 | 601530 | GTR000523316.8 | Amyotrophic Lateral Sclerosis and Frontotemporal Dementia |
| TARDBP | 1p36.22 | 605078 | GTR000523316.8 | Amyotrophic Lateral Sclerosis and Frontotemporal Dementia |
| TREM2 | 6p21.1 | 605086 | GTR000523316.8 | Amyotrophic Lateral Sclerosis and Frontotemporal Dementia |
| UBQLN2 | Xp11.21 | 300264 | GTR000523316.8,GTR000521117.2 | Amyotrophic Lateral Sclerosis and Frontotemporal Dementia,Hereditary Spastic Paraplegia |
| VAPB | 20q13.32 | 605704 | GTR000523316.8 | Amyotrophic Lateral Sclerosis and Frontotemporal Dementia |
| VCP | 9p13.3 | 601023 | GTR000523316.8 | Amyotrophic Lateral Sclerosis and Frontotemporal Dementia |
| AFG3L2 | 18p11.21 | 604581 | GTR000531634.1,GTR000523795.1 | Hereditary ataxias |
| ANO10 | 3p22.1-21.33 | 613726 | GTR000531634.1,GTR000523795.1 | Hereditary ataxias |
| APTX | 9p21.1 | 606350 | GTR000531634.1,GTR000523795.1 | Hereditary ataxias |
| ATM | 11q22.3 | 607585 | GTR000531634.1,GTR000523795.1 | Hereditary ataxias |
| CACNA1A | 19p13.13 | 601011 | GTR000531634.1,GTR000523795.1 | Hereditary ataxias |
| CACNB4 | 2q23.3 | 601949 | GTR000531634.1,GTR000523795.1 | Hereditary ataxias |
| COQ8A | 1q42.13 | 606980 | GTR000531634.1,GTR000523795.1 | Hereditary ataxias |
| FGF14 | 13q33.1 | 601515 | GTR000531634.1,GTR000523795.1 | Hereditary ataxias |
| FXN | 9q21.11 | 606829 | GTR000531634.1,GTR000523795.1 | Hereditary ataxias |
| IFRD1 | 7q31.1 | 603502 | GTR000531634.1 | Hereditary ataxias |
| ITPR1 | 3p26.1 | 147265 | GTR000531634.1,GTR000523795.1 | Hereditary ataxias |
| KCNA1 | 12p13.32 | 176260 | GTR000531634.1,GTR000523795.1 | Hereditary ataxias |
| KCNC3 | 19q13.33 | 176264 | GTR000531634.1,GTR000523795.1 | Hereditary ataxias |
| MRE11A | 11q21 | 600814 | GTR000531634.1,GTR000523795.1 | Hereditary ataxias |
| MTPAP | 10p11.23 | 613669 | GTR000531634.1,GTR000523795.1 | Hereditary ataxias |
| MTTP | 4q23 | 157147 | GTR000531634.1,GTR000523795.1 | Hereditary ataxias |
| PDYN | 20p13 | 131340 | GTR000531634.1,GTR000523795.1 | Hereditary ataxias |
| PIK3R5 | 17p13.1 | 611317 | GTR000531634.1,GTR000523795.1 | Hereditary ataxias |
| POLG | 15q26.1 | 174763 | GTR000531634.1,GTR000523795.1 | Hereditary ataxias |
| PRKCG | 19q13.42 | 176980 | GTR000531634.1,GTR000523795.1 | Hereditary ataxias |
| SACS | 13q12.12 | 604490 | GTR000531634.1,GTR000523795.1,GTR000514976.2,GTR000521117.2 | Hereditary ataxias,Hereditary Spastic Paraplegia |
| SETX | 9q34.13 | 608465 | GTR000531634.1,GTR000523795.1 | Hereditary ataxias |
| SIL1 | 5q31.2 | 608005 | GTR000531634.1,GTR000523795.1 | Hereditary ataxias |
| SLC1A3 | 5p13.2 | 600111 | GTR000531634.1,GTR000523795.1 | Hereditary ataxias |
| SPTBN2 | 11q13.2 | 604985 | GTR000531634.1,GTR000523795.1 | Hereditary ataxias |
| SYNE1 | 6q25.2 | 608441 | GTR000531634.1,GTR000523795.1 | Hereditary ataxias |
| SYT14 | 1q32.2 | 610949 | GTR000531634.1,GTR000523795.1 | Hereditary ataxias |
| TDP1 | 14q32.11 | 607198 | GTR000531634.1,GTR000523795.1 | Hereditary ataxias |
| TGM6 | 20p13 | 613900 | GTR000531634.1,GTR000523795.1 | Hereditary ataxias |
| TTBK2 | 15q15.2 | 611695 | GTR000531634.1,GTR000523795.1 | Hereditary ataxias |
| TTPA | 8q12.3 | 600415 | GTR000531634.1,GTR000523795.1 | Hereditary ataxias |
| ZNF592 | 15q25.3 | 613624 | GTR000531634.1,GTR000523795.1 | Hereditary ataxias |
| ABCB7 | Xq13.3 | 300135 | GTR000523795.1 | Hereditary ataxias |
| ABHD12 | 20p11.21 | 613599 | GTR000523795.1 | Hereditary ataxias |
| CCDC88C | 14q32.11-32.12 | 611204 | GTR000523795.1 | Hereditary ataxias |
| DNMT1 | 19p13.2 | 126375 | GTR000523795.1 | Hereditary ataxias |
| EEF2 | 19p13.3 | 130610 | GTR000523795.1 | Hereditary ataxias |
| ELOVL4 | 6q14.1 | 605512 | GTR000523795.1 | Hereditary ataxias |
